# Cajal’s contributions to vestibular research

**DOI:** 10.3389/fnana.2024.1476640

**Published:** 2024-09-17

**Authors:** Juan M. Espinosa-Sanchez, Nicolas Perez-Fernandez, Fernando de Castro, Angel Batuecas-Caletrio

**Affiliations:** ^1^Division of Otoneurology, Department of Otolaryngology, Hospital Universitario Virgen de las Nieves, Granada, Spain; ^2^Division of Otolaryngology, Department of Surgery, University of Granada, Granada, Spain; ^3^Otology and Neurotology Group CTS495, Instituto de Investigación Biosanitaria ibs.GRANADA, Granada, Spain; ^4^Sensorineural Pathology Programme, Centro de Investigación Biomédica en Red en Enfermedades Raras, CIBERER, Madrid, Spain; ^5^Department of Otorhinolaryngology, Clínica Universidad de Navarra, Madrid, Spain; ^6^Instituto Cajal-CSIC, Consejo Superior de Investigaciones Científicas-CSIC, Madrid, Spain; ^7^Otoneurology Unit, ENT Department, University Hospital of Salamanca, IBSAL, Salamanca, Spain; ^8^Surgery Department, Faculty of Medicine, University of Salamanca, Salamanca, Spain

**Keywords:** Cajal, vestibular system, history of neuro-otology, history of neuroanatomy, history of vestibular medicine, Retzius, Bárány

## Abstract

The Spanish neurohistologist Santiago Ramón y Cajal (1852–1934) is widely regarded as the father of modern Neuroscience. In addition to identifying the individuality of cells in the nervous system (the neuron theory) or the direction followed by nerve impulses (the principle of dynamic polarization), he described numerous details regarding the organization of the different structures of the nervous system. This task was compiled in his magnum opus, “Textura del Sistema Nervioso del Hombre y los Vertebrados,” first published in Spanish between 1899 and 1904, and later revised and updated in French as “Histologie du système nerveux de l’homme et des vertébrés” between 1909 and 1911 for wider distribution among the international scientific community. Some of Cajal’s findings are fundamental to our understanding of the anatomy and histology of the vestibular system. He depicted the nerve endings in the sensory epithelia, the structure of the vestibular nerve and Scarpa ganglion, afferent vestibular fibers, vestibular nuclei, lateral vestibulospinal tract, vestibulocerebellar connections, and the fine structure of the cerebellum. However, most of these pioneering descriptions were published years earlier in Spanish journals with limited circulation. Our study aimed to gather Cajal’s findings on the vestibular system and identify his original publications. After this endeavor, we claim a place for Cajal among the founders of anatomy and histology of the vestibular system.

## Introduction

**Figure 1:**
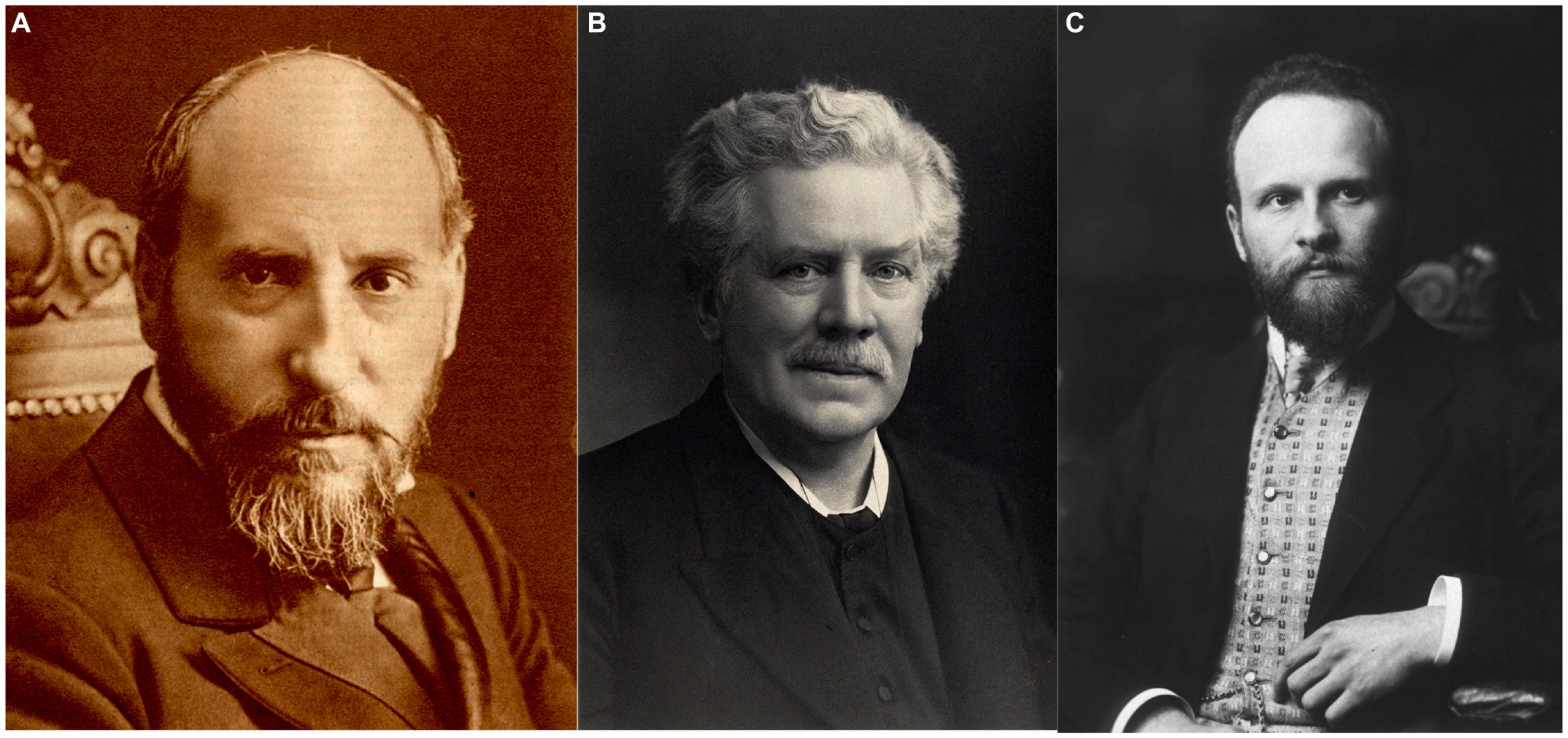
The main characters of the subject. **(A)** Santiago Ramón y Cajal (1852–1934) [from Banco de imágenes de la Medicina Española]. **(B)** Gustav Retzius (1842–1919) [from Wellcome Library-London, UK-, copyrighted work available under Creative Commons Attribution only licence CC BY 4.0]. **(C)** Robert Barany (1876–1936) [public domain].

Santiago Ramón y Cajal (1852–1934; Figure 1A) is widely recognized as the father of modern neuroscience (DeFelipe, 2002). In 1888, he provided the first scientific evidence for the intuitive free-ending hypothesis (Ramón y Cajal, 1888a, 1888b) originally proposed by Wilhelm His (1831–1904) and Auguste-Henri Forel (1848–1931). Thus, he extended to the nervous system the cell theory initially formulated by Matthias Jacob Schleiden (1804–1881) and Theodor Schwann (1810–1882), and later developed by Rudolf Virchow (1821–1902). Cajal claimed that neurons are the fundamental anatomical and functional units of the nervous system, a concept that soon became the neuron doctrine–an absolute foundational milestone in neuroscience (Shepherd, 2016). He stated that nerve impulses are transmitted from one neuron to another through contact rather than contiguity, thereby refuting the reticular theory proposed by Joseph von Gerlach (1820–1896) and supported by Camillo Golgi (1844–1926). Additionally, Cajal formulated the principle of dynamic polarization in 1889, which established that nerve impulses are transmitted from the dendrites and cell body towards the axon, reaching the dendrites of the neurons behind the synaptic cleft (Ramón y Cajal, 1889). He also described the dendritic spines (Ramón y Cajal, 1888a) and the axonal growth cones (Ramón y Cajal, 1890a, 1890b), assigning them specific role on the functioning and formation of synapses. Here, we must point out that Cajal called “interneuronal articulations” to what we know today as synapses, a term originally introduced by Sherrington (1897), and which Cajal never employed. It was not until more than 50 years later that Palay (1956) confirmed their existence through electron microscopy and introduced the term synaptic cleft. Furthermore, Cajal proposed the chemotactic or, in his own words, “neurotropic hypothesis” on how axonal growth cones should be guided up to their targets to form synapses (Ramón y Cajal, 1892b, 1899–1904). In the last period of his scientific work, Cajal focused on the degeneration and regeneration of the nervous system (Ramón y Cajal, 1913–1914) and never abandoned his fight against reticularists (Ramón y Cajal, 1933).

Although he studied almost every structure in the nervous system, Cajal devoted special dedication to the microscopic anatomy of the cerebellum, retina, and cerebral cortex. These findings and many others were all compiled in his *magnum opus*: *Textura del Sistema Nervioso del Hombre y de los Vertebrados* (Ramón y Cajal, 1899–1904), first published in Spanish and subsequently improved and translated into French as *Histologie du système nerveux de l’homme et des vertébrés* (Ramón y Cajal, 1909–1911). This study is still considered among the most comprehensive compilations of the microscopic anatomy of the nervous system. Providing a version with updated and accurate nomenclature of structures and cell types would greatly enhance its usefulness for both current and future generations of neuroscientists.

Cajal’s contributions to the understanding of the anatomy and histology of the vestibular system are often overlooked due to the majority of his work not being published in English. This has led to his authorship being underappreciated or misrepresented through incorrect citations. The aim of this study was to identify, systematize and highlight these significant contributions, while also noting the original works in which Cajal first described his findings. To achieve this goal, the entire body of Cajal’s work was carefully reviewed, and his original drawings, which accompanied his findings, are presented.

### Anatomy and histology of the vestibular system before Cajal

The knowledge of the anatomy of the inner ear or labyrinth was already well established at the beginning of the 19th century (Hawkins, 2004). The semicircular canals were precisely described by Gabriele Falloppio (c. 1523–1562) in his work *Observationes Anatomicae* (Falloppio, 1561), while Bartolomeo Eustachio (c. 1520–1574) delved deeper into the structure that bears his name, as well as the tensor tympani and stapedial muscles, in his *Epistula de Auditus Organis* (Eustachio, 1564).

During the 19th century, the histology of the inner ear was studied in depth. Alfonso Corti (1822–1876), while working in Albert von Kölliker’s laboratory at the University of Würzburg, provided detailed descriptions of the sensory epithelium of the cochlea, spiral ganglion, tectorial membrane, and stria vascularis (Corti, 1851). Between 1881 and 1884, Gustaf Magnus Retzius (1842–1919), professor of Anatomy at the Karolinska Institute, published two significant works on the inner ear which examined the comparative anatomy of the inner ear in vertebrates (Retzius, 1881a; Retzius, 1882; Retzius, 1884; for a specific historical review, see: Hawkins, 2005; Yaşargil and Yaşargil, 2016). Retzius thoroughly described the contact between the fibers of cranial nerve VIII and the basal end of the hair cells of the sensory organs.

Cajal, who had previously exchanged correspondence with Retzius, first meet the influential Swedish researcher in Berlin at the congress of the German Anatomical Society in 1899. From that point on, Retzius, along with Kölliker, became one of the main supporters of the neuron theory. Cajal sustained a prolonged scientific and friendly relationship with Retzius in such a way that Retzius, alongside Rafael Lorente de Nó, was the principal recipient of Cajal’s correspondence preserved to date (Fernández Santarén, 2014).

Schultze (1858) was the first author to mention the sensory epithelium of the cristae ampullares and the otolithic maculae. Later, Odenius (1867), Hasse (1867) and Retzius (1881b, 1884, 1892) made further contributions. Hans Held (1866–1942) discussed the bipolar nature of neurons in the vestibular ganglion and identified two types of cells in the ampullary crests: sensory and supporting cells.

Over the course of the 19th century, several other researchers, including Friedrich Christian Rosenthal (1780–1829), Ernst Reissner (1824–1878), Friedrich Matthias Claudius (1822–1869), Otto Deiters (1834–1863), Viktor Hensen (1835–1924), and Jean-Pierre Nuel (1847–1920), also made substantial contributions to our understanding of the histology of the inner ear (for specific reviews on these, see: Mudry, 2001; Schacht and Hawkins, 2004).

## Cajal’s contributions to the anatomy and histology of the vestibular system

The vestibular system is responsible for the maintenance of balance and posture. The vestibular labyrinth comprises five specific sensory organs, which include three cristae ampullares (horizontal, anterior, and posterior) situated within the semicircular canals, and the maculae of the utricle and saccule. The semicircular canals sense angular accelerations whereas the two otolith organs are sensitive to linear accelerations. Afferent sensory signals from the cristae and maculae are transmitted by the vestibular nerve. It enters the brainstem at the ventrolateral aspect of the pontomedullary junction and then projects to the vestibular nuclei of the brainstem and cerebellum. In the CNS, this sensory information is integrated with inputs from visual and proprioceptive afferents, and is processed and modulated to produce two reflexes: the vestibulo-ocular reflex, which is responsible for gaze stabilization, and the vestibulo-spinal reflex, which is involved in postural control, thus achieving the perception of balance and posture.

Golgi discovered a method for microscopic visualization of nerve cells using silver nitrate after tissue fixation in potassium dichromate in 1873. Cajal utilized Golgi’s method extensively, but he made modifications to it by employing the double silver impregnation technique (Ramón y Cajal, 1889). These metal impregnation techniques, alongside other procedures developed by Cajal and his students, the so-called Spanish Neurohistological School (Ramón y Cajal and De Castro Rodríguez, 1933; Merchán et al., 2016; de Castro, 2019), were of prime importance for his detailed description of the microscopic anatomy of the vestibular system.

### Vestibular sensory epithelia

As we have noted, Retzius examined how the fibers of the cochleo-vestibular nerve reached the hair cells of the sensory organs of the inner ear through a series of papers released between 1871 and 1884. In 1892, using Cajal’s method of double silver impregnation, Retzius depicted the peripheral endings of the vestibular nerve freely ending on the vestibular sensorial epithelium (Retzius, 1892). The following year, Cajal confirmed these findings on the innervation of the “acoustic crests,” which was the term then used for cristae ampullares. He subsequently communicated it to Retzius in a letter in which he also thanked him for sending “his groundbreaking work on the auditory system of vertebrates,” acknowledging that “my finances would not have permitted me to acquire such a significant work.”^1^

Ramón y Cajal (1892a, p. 529–530) used these results to reaffirm both the neural theory and the principle of dynamic polarization. However, Held (1902) and Kolmer (1904), advocates of the reticular theory, argued that this contact was an artifact. The controversy regarding the presence or absence of cell continuity between the nervous endings and hair cells was finally clarified by Rafael Lorente de Nó (1902–1990), the last of the direct disciples of Cajal (Lorente de Nó, 1926).

Cajal described the sensory epithelium of cristae and maculae distinguishing between hair cells and supporting cells (Ramón y Cajal, 1904). He depicted hair cells as cylindrical and shorter than supporting cells, with their deep part rounded and slightly thickened, containing the nucleus. Cajal noted that the superficial end is covered with a cuticle, giving rise to a large, rigid cilium that is bathed in the endolymph and contacted by special calcium carbonate crystals found in the labyrinth of all vertebrates. He also suggested that the cilia of neuroepithelial cells appear to be stimulated when the endolymph is shifted by head movements. It should also be noted that Cajal appointed cristae ampullares to lack otoliths, whereas otolithic maculae possess them in abundance (Ramón y Cajal, 1908e). However, Cajal did not differentiate hair cell types, so it had to be Jan Wersäll’s electron microscopy work in the 1950s that provided the contemporary description of type I and type II hair cells (Wersäll, 1954; Wersäll, 1956; Engström and Wersäll, 1958).

**Figure 2:**
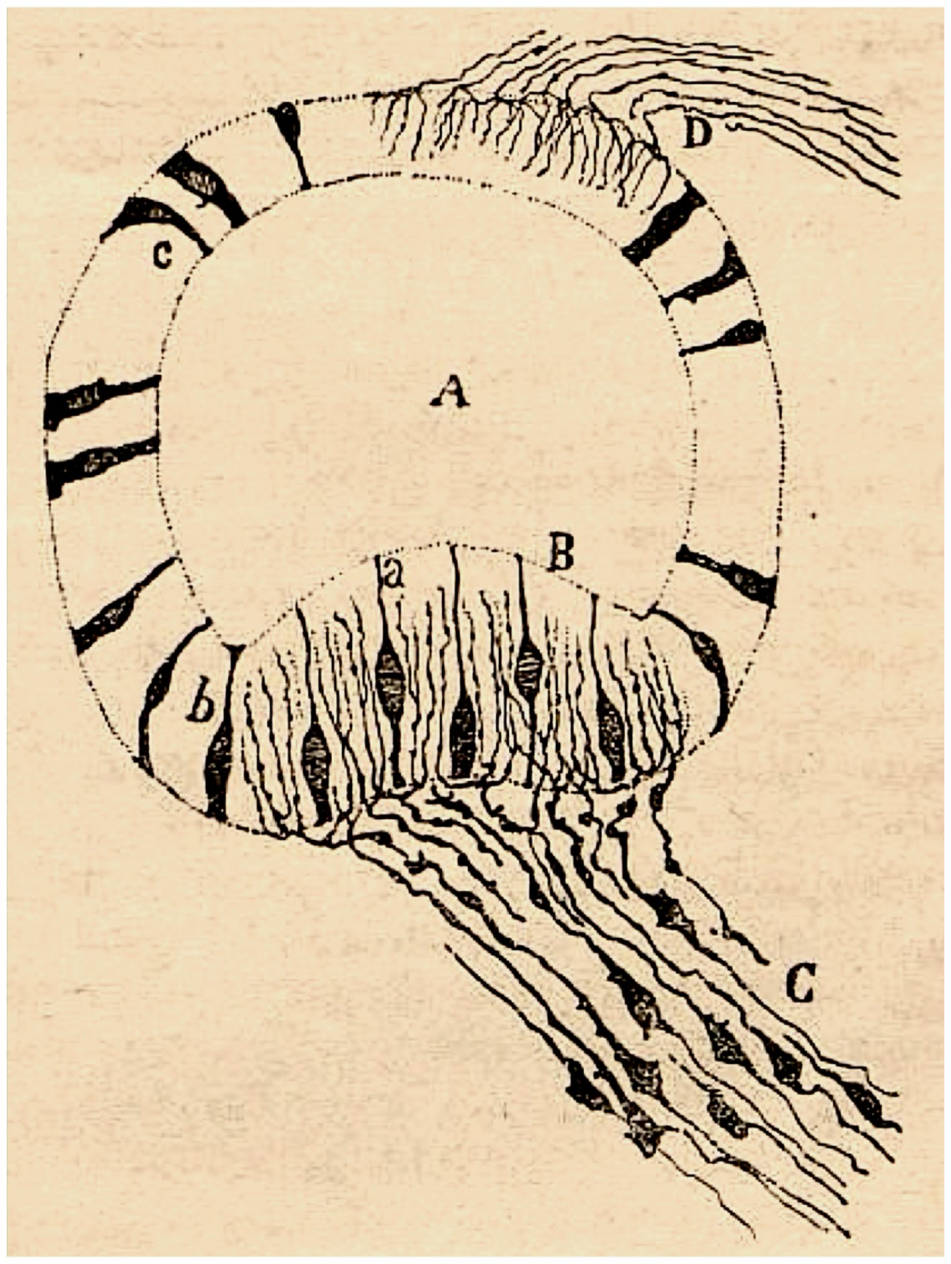
Crista ampullaris of a mouse embryo (transversal section). *(A)* semicircular canal; *(B)* crista ampullaris; *(C)* afferent fibers; *(a–c)* sensory cells (reproduced from Ramón y Cajal, 1892a, Figure 21; also appeared as Figure 30 in Ramón y Cajal, 1894).

**Figure 3:**
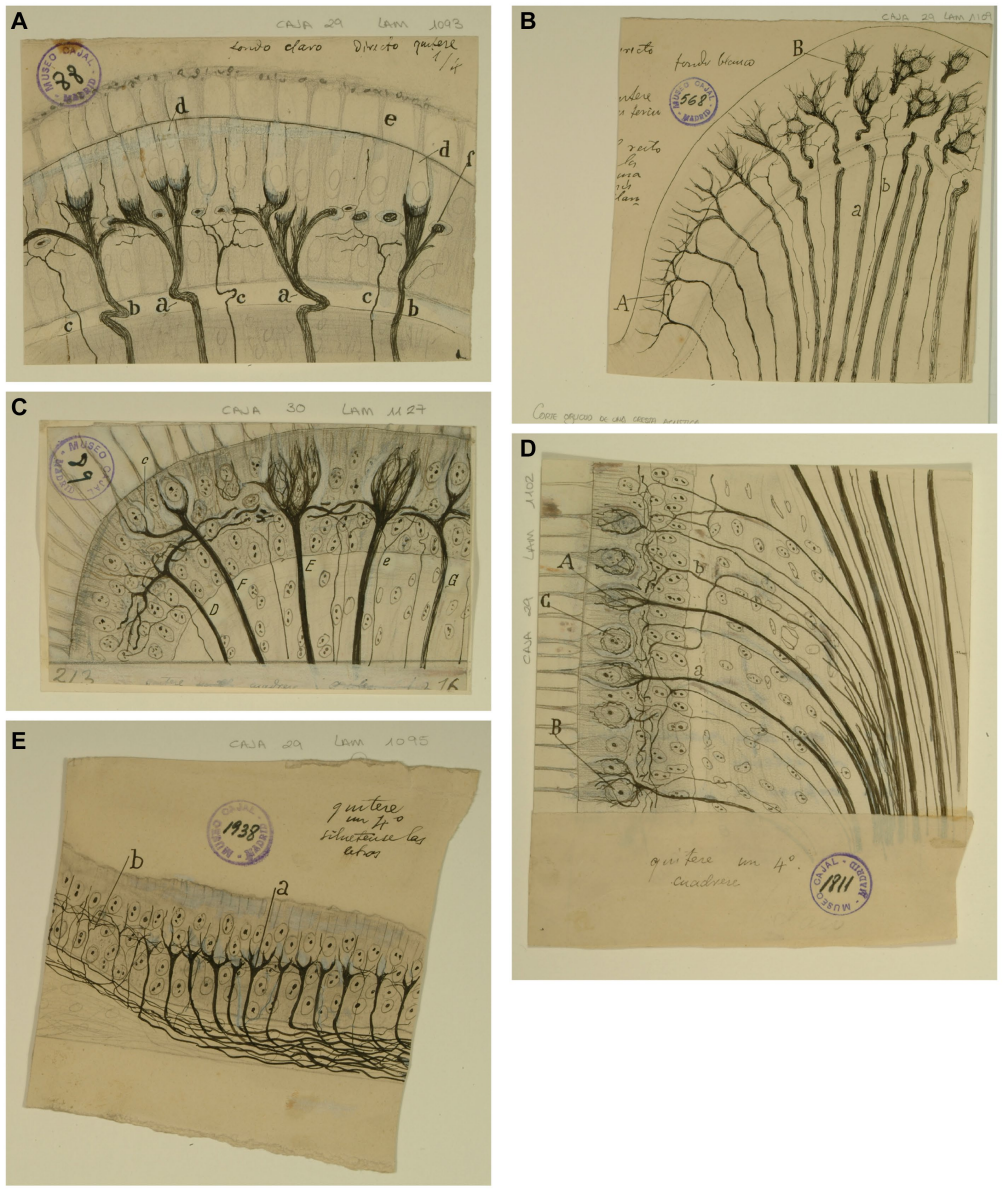
Types of afferent endings of vestibular nerve in birds and fishes. **(A)** Crista ampullaris of a chick embryo. *(a,b)* Calyceal endings from colossal (giant) fibers; *(c)* fine fibers; *(d)* hair cells. [Cajal’s original drawing, from Legado Cajal-CSIC,. Madrid; first published in Ramón y Cajal, 1904]. **(B)** Oblique section of a crista ampullaris (E16 chick embryo, reduced silver nitrate technique). *(A)* Region where the thin fibers end; *(B)* region where the colossal fibers end; *(a)* colossal fibers that end in calyces; *(b)* thin fibers [Cajal’s original drawing, from Legado Cajal-CSIC, Madrid; first published in Ramón y Cajal, 1904, Figure 9; it was also published as Figure 317 in Ramón y Cajal, 1909–1911, vol. 1]. **(C)** Vestibular endings in a crista ampullaris (few days bird). *(E)* colossal fiber to several hair cells; *(F)* another fiber that generates two terminal nests; *(D)* fiber ending in an intraepithelial horizontal plexus. [Cajal’s original drawing, from Legado Cajal-CSIC, Madrid; first published in Ramón y Cajal, 1908e, Figure 7]. **(D)** Section of the utricular macula (few days sparrow). *(A)* Pericellular nests formed by medium thickness fibers; *(B)* Another fiber that generates two nests; *(C)* obliquely cut hair cell; *(b)* fine fiber to the intraepithelial horizontal plexus. [Cajal’s original drawing, from Legado Cajal-CSIC, Madrid; first published in Ramón y Cajal, 1908e, Figure 8]. **(E)** Endings of the vestibular nerve in the macula (E14 trout embryo) *(a)* final branches of the colossal fibers; *(b)* fine fibers branches [Cajal’s original drawing, from Legado Cajal-CSIC, Madrid; first published in Ramón y Cajal, 1908d, Figure 3].

**Figure 4:**
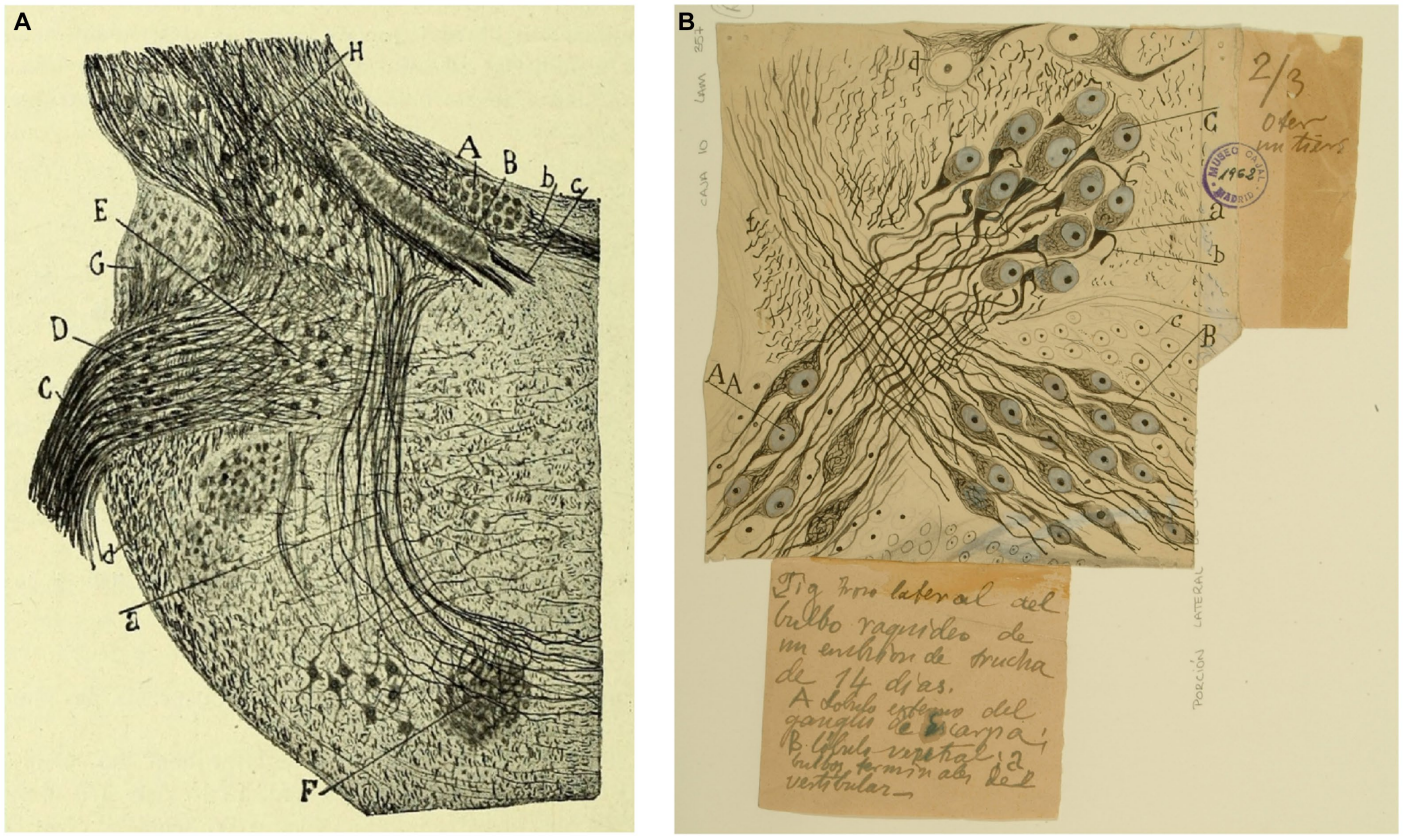
Tangential vestibular nucleus in birds and fishes. **(A)** Section of the acoustic region in birds. (Medulla of a few-days-old kite. *Milvus regalis*, Briss. Reduced silver nitrate method). *(A)* Magnocellular nucleus; *(B)* parvocellular nucleus; *(C)* vestibular root of the VIII nerve; *(D)* tangential or interstitial nucleus; *(E)* lateral (Deiters´) vestibular nucleus; *(F)* superior olive; *(a)* trapezoid body [reproduced from Ramón y Cajal, 1908d, Figure 1; also appeared in Ramón y Cajal, 1909–1911, vol. 1, Figure 365]. **(B)** Lateral part of the medulla oblongata (E14 trout embryo). *(A)* External part of the Scarpa’s ganglion, which gives rise to the colossal fibers of the tangential nucleus; *(B)* ventral part of the Scarpa’s ganglion; *(C)* tangential nucleus; *(b)* descending branch from the vestibular nerve; neurons from Scarpa’s ganglion [Cajal’s original drawing, from Legado Cajal-CSIC, Madrid; first published in Ramón y Cajal, 1908d, Figure 4. The handwritten notes on the right and bottom are by Cajal himself, giving instructions to the printer to obtain the best quality of reproduction].

He also depicted the afferent endings of the bipolar neurons of the Scarpa ganglion in the cristae ampullares of the semicircular canals and in the maculae of the otolith organs (Ramón y Cajal, 1892a, pp. 529–530, 1894, pp. 127–130; Figure 2). Regarding the afferent innervation patterns, he demonstrated two types of nerve fibers in birds and fishes: colossal (giant) fibers and fine fibers (Ramón y Cajal, 1904, 1908d, 1908e; Figure 3). According to Cajal, the first ones end in one or more hair cells by means of calyceal endings which extend in multitude of ascending twigs surrounding the hair cell. Furthermore, the colossal fibers emit thick horizontal branches that extend beneath the hair cells. On the other hand, fine fibers branch out diffusely to form a plexus. The colossal fibers extend preferentially in the summit and lateral zone of the cristae, while the fine fibers are found in the peripheral zone (Figure 3A). These studies, together with the cited one from Lorente de Nó (1926), paved the work of César Fernández, Jay M. Goldberg and Richard A. Baird in the late 1980s that gave rise to the current morphological classification of afferent endings into three categories: calyceal, bouton, and dimorphic (Fernandez et al., 1988; Fernandez et al., 1990). The colossal fibers were also described by Cajal in the tangential vestibular nucleus of the medulla oblongata of the chick, where they form large axosomatic synapses *en passant* or spoon endings on the principal cell bodies (Ramón y Cajal, 1908d; Figure 4).

### Scarpa’s ganglion and vestibular nerve

**Figure 5:**
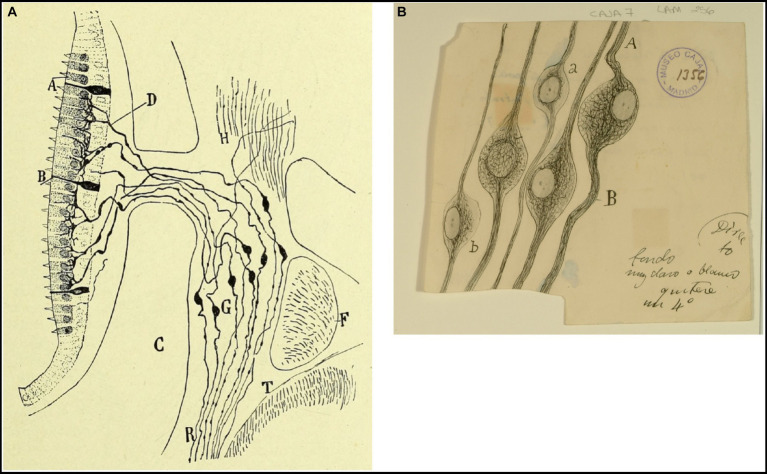
Vestibular ganglion in mammals and birds. **(A)** Vestibular (or Scarpa’s) ganglion and peripheral endings of the vestibular nerve in the macula (near-term mouse fetus, Golgi method). *(A)* Hairs cells; *(B)* supporting cells; *(C)* temporal bone; *(D)* preterminal bifucations of the peripheral branches; *(F)* facial nerve; *(G)* Scarpa’s ganglion; *(T)* medulla oblongata [reproduced from Ramón y Cajal, 1899–1904, vol. 2, Figure 245]. **(B)** Neurons from the vestibular (Scarpa’s) ganglion in the chick embryo [Cajal’s original drawing, from Legado Cajal-CSIC, Madrid; first published in Ramón y Cajal, 1904].

The cell bodies of first-order vestibular neurons constitute the Scarpa’s ganglion, located at the fundus of the internal auditory meatus. Taking into consideration the previous works of His (1889), Retzius (1892) and Von Lenhossék (1894), Cajal studied the structure of the Scarpa’s ganglion (Ramón y Cajal, 1899–1904, p. 89–94; Figure 5). However, he failed to comprehend how the ganglion is divided into an upper and a lower portion. This task would be carried out once again by his disciple Lorente de Nó, who would further differentiate in Scarpa’s ganglion five parts based on their cytoarchitecture, and that would correspond to the five vestibular receptors. Lorente de Nó, in accordance with Voit (1907), detailed systematically that the fibers of the superior vestibular nerve provide innervation to the cristae ampullares of the anterior and horizontal ducts, as well as the macula of the utricle. On the other hand, the inferior branches supply the crista of the posterior canal, while the macula of the saccule receives innervation from both nerves (Lorente de Nó, 1926, 1931).

Ramón y Cajal (1899–1904, p. 94) appreciated inside the ganglion of Scarpa fibers from the nerve of Wrisberg and suggested that they could be sympathetic fibers or sensitive branches of the geniculate ganglion. Using electron microscopy, Wersäll (1956) confirmed the existence of unmyelinated fibers in the vestibular nerve.

**Figure 6:**
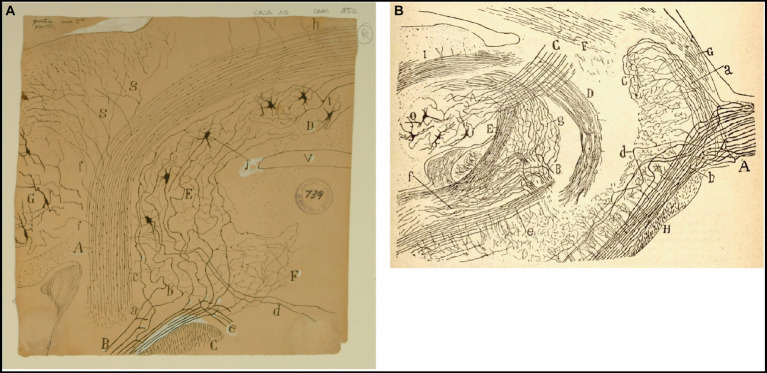
Cajal on the vestibular nuclei. **(A)** Frontal section taken through the pons and cerebellum (newborn mouse, Golgi method). *(A)* Inferior cerebellar peduncle; *(B)* roots of the vestibular nerve; *(C)* spinal root of the trigeminal nerve; *(D)* fastigial nucleus; *(E)* superior (Betchterew) vestibular nucleus; *(F)* rostral end of the lateral (Deiters*´*) vestibular nucleus; *(G)* dentate nucleus; *(a)* ascending branch of vestibular nerve; *(b)* descending branch of vestibular nerve [Cajal’s original drawing, from Legado Cajal-CSIC, Madrid; first published in Ramón y Cajal, 1895b, Figure 18; it was also published as Figure 18 in Ramón y Cajal, 1896 and Figures 248 and 444 in Ramón y Cajal, 1899–1904]. **(B)** Very lateral sagittal section of the pons and cerebellum (mouse fetus, Golgi method). *(A)* Sensitive root of the trigeminal nerve with *(a)* ascending and *(b)* descending branches of the trigeminal nerve; *(c)* terminal arborization of ascending branches; *(e)* posterior part of the descending sensitive root; *(B)* bifurcation of the vestibular nerve with *(g)* ascending branches towards the cerebellum, and *(f)* descending branches towards the medulla oblongata; *(C)* superior cerebellar peduncle; *(D)* uncrossed dentato-bulbar tract; *(E)* inferior cerebellar peduncle; *(F)* lateral lemniscus; *(G)* middle cerebellar pedundle; *(H)* trapezoid body; *(O)* dentate nucleus [reproduced from Ramón y Cajal, 1895b, Figure 1; also appeared as Figure 1 in Ramón y Cajal, 1896 and as Figure 433 in Ramon y Cajal, 1899–1904, vol. 2].

Cajal not only described the peripheral processes of the bipolar sensory neurons in the vestibular ganglion but also their central or proximal processes that form the vestibular nerve entering the medulla between the ventral aspect of the inferior cerebellar peduncles and the dorsal aspect of the spinal root of the trigeminal nerve (Ramón y Cajal, 1895b, p. 59–72; Figure 6A). He noted that these fibers are the main afferents of the vestibular nuclei; however, some of them directly pass to the cerebellum via the inferior cerebellar peduncle forming the direct vestibulocerebellar tract.

**Figure 7:**
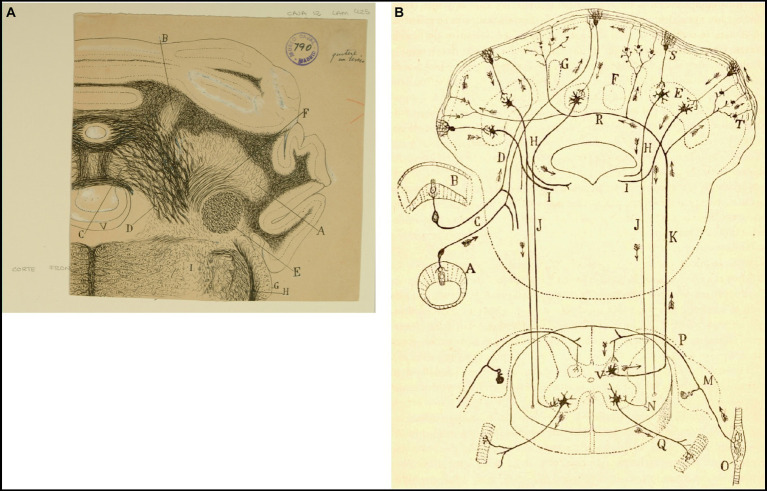
How Cajal saw the connectivity between the vestibular system and cerebellum. **(A)** Frontal section through the medulla oblongata and cerebellum of the guinea pig (Weigert-Pal and carmine method). *(A)* Dentate nucleus; *(B)* emboliform nucleus; *(C)* fastigial nucleus; *(D)* cerebellovestibular nucleus and vestibulo-cerebellar bundle; *(E)* inferior cerebellar peduncle; *(F)* superior cerebellar peduncles; *(G)* dorsal cochlear nucleus; *(H)* dorsal part of trigeminal root; *(V)* fourth ventricule [Cajal’s original drawing, from Legado Cajal-CSIC, Madrid; first published as Figure 425 in Ramón y Cajal, 1899–1904, vol. 2]. **(B)** Diagram of the afferent and efferent pathways of the cerebellum (Arrows indicate the direction of current flow). *(A)* Antero-posterior semicircular conduct; *(B)* transversal semicircular conduct; *(C)* vestibular nerve; *(D)* ascending branch of vestibular nerve connecting with the cerebellum; *(E)* dentate nucleus; *(H)* superior cerebellar peduncle; *(I)* uncrossed (direct) descending branch of the superior cerebellar peduncle; *(J)* crossed descending branch of the superior cerebellar peduncle; *(K)* ascending sensory pathway arising in Clarke’s column that may synapse with granule cells by way of mossy fibers; *(M)* spinal ganglion; *(N)* termination of the crossed descending branch of the superior cerebellar peduncle in the ventral horn of the spinal cord; *(O)* Kühne muscle spindle; *(P)* dorsal root synapsing in Clarke’s column; *(Q)* ventral or motor root; *(R)* dorsal spinocerebellar tract; *(S)* Purkinje cell; *(T)* mossy sibers; *(V)* Clarke’s column giving rise to the dorsal spinocerebellar tract [reproduced from Ramón y Cajal, 1899–1904, vol. 2, Figure 445; we use here the caption from Ramón y Cajal, 1909–1911, vol. 2, Figure 101].

Using the Golgi method, Cajal demostrated the course of the fibers of the vestibular nerve toward the vestibular nuclei and cerebellum after leaving the inner auditory meatus. He expanded on the descriptions of Kölliker (1891) and Held (1892) describing how the vestibular nerve penetrates the brainstem at the pontomedullary junction and splits into an ascending branch and a descending branch (Ramón y Cajal, 1895b, pp. 59–65; 1896, pp. 61–67; Figure 6). According to Cajal, the ascending branch winds rostrally and dorsally to enter the superior nucleus and joins the vestibulo-cerebellar bundle (referred to by Cajal as the acoustic-cerebellar bundle). It sends numerous collaterals to the rostral portion of the lateral vestibular nucleus and mostly to the superior nucleus, and finally to the ipsilateral fastigial nucleus and, bilaterally, to the cerebellar vermis. Ramón y Cajal (1895b, pp. 59–72) was also the first author to describe the existence of primary vestibulocerebellar fibers that reach the cerebellum passing through the medial portion of the inferior cerebellar peduncle (juxtarestiform body) without making synapses in the vestibular nuclei (Figure 6A). Notably, this finding, later confirmed by Brodal and Høivik (1964), was denied by Lorente de Nó (1924, 1933a). Ramón y Cajal (1899–1904) also specified that these primary vestibular afferent projections to the cerebellum terminate in the cerebellar vermis and the fastigial nucleus by means of mossy fibers (Figures 6A, 7).

On the other hand, Cajal identified a descending portion, thicker, that reaches the descendent nucleus. At present, we know that the rostral branch also projects to the medial nucleus and that the caudal branch also reaches the lateral vestibular nucleus (Vidal et al., 2015).

### Vestibular nuclei

**Figure 8:**
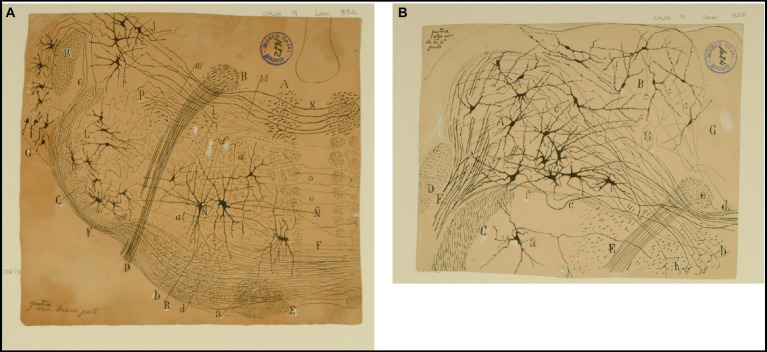
The lateral (direct) and medial (crossed) vestibular pathway. **(A)** Frontal section through the medulla oblongata at the level of the facial nerve and trapezoid body (newborn mouse, Golgi technique). *(A)* MLF containing fibers from the crossed vestibular pathway entering the MLF at this level; *(B)* facial nerve, *(C)* trigeminal nerve (spinal root); *(D)* exit of the facial nerve; *(E)* pyramidal tract; *(F)* ventral trapezoid fibers; *(G)* ventral cochlear nucleus; *(H)* dorsal cochlear nucleus; *(J)* lateral (Deiters′) vestibular nucleus; *(L)* spinal nucleus of the trigeminal nerve; *(M)* abducens nucleus with collaterals from the MLF; *(N)* axons from the lateral (Deiters*´*) vestibular nucleus forming the crossed secondary vestibular pathway; *(O)* trapezoid fibers from the dorsal cochlear nucleus; *(P)* uncrossed trigeminal pathway. [Cajal’s original drawing, from Legado Cajal-CSIC, Madrid; this important illustration was first published in Ramón y Cajal, 1895b, Figures 3 and 15, but was also later included in Ramón y Cajal, 1899–1904, vol. 2, where it appeared three times as Figures 254, 284, and 304]. **(B)** Frontal section through dorsolateral parts of the medulla oblongata (newborn mouse, Golgi tecnique). *(A)* Lateral (Deiters´) vestibular nucleus; *(B)* medial (principal) vestibular nucleus; *(C)* spinal root of the trigeminal nerve; *(D)* restiform body; *(E)* facial nerve; *(F)* vestibular nerve roots; *(G)* gray central substance; *(a)* neuron in the spinal (gelatinous) nucleus of the trigeminal nerve; *(b´)* ipsilateral (uncrossed) second order (lateral) vestibular pathway; *(d´)* crossed second-order (medial) vestibular pathway reaching the raphe; *(a)* ascending and *d)* descending axons in the ipsilateral vestibular pathway; *(c)* axons; *(e)* the knee of the facial nerve; *(f)* vestibular fibres (apparently, searching the raphe); *(g)* axon collaterals coming from the lateral vestibular nucleus. [Cajal’s original drawing, from Legado Cajal-CSIC, Madrid; first published in Ramón y Cajal, 1895b, Figure 19, later included in Ramón y Cajal, 1899–1904, vol. 2, Figure 250].

**Figure 9:**
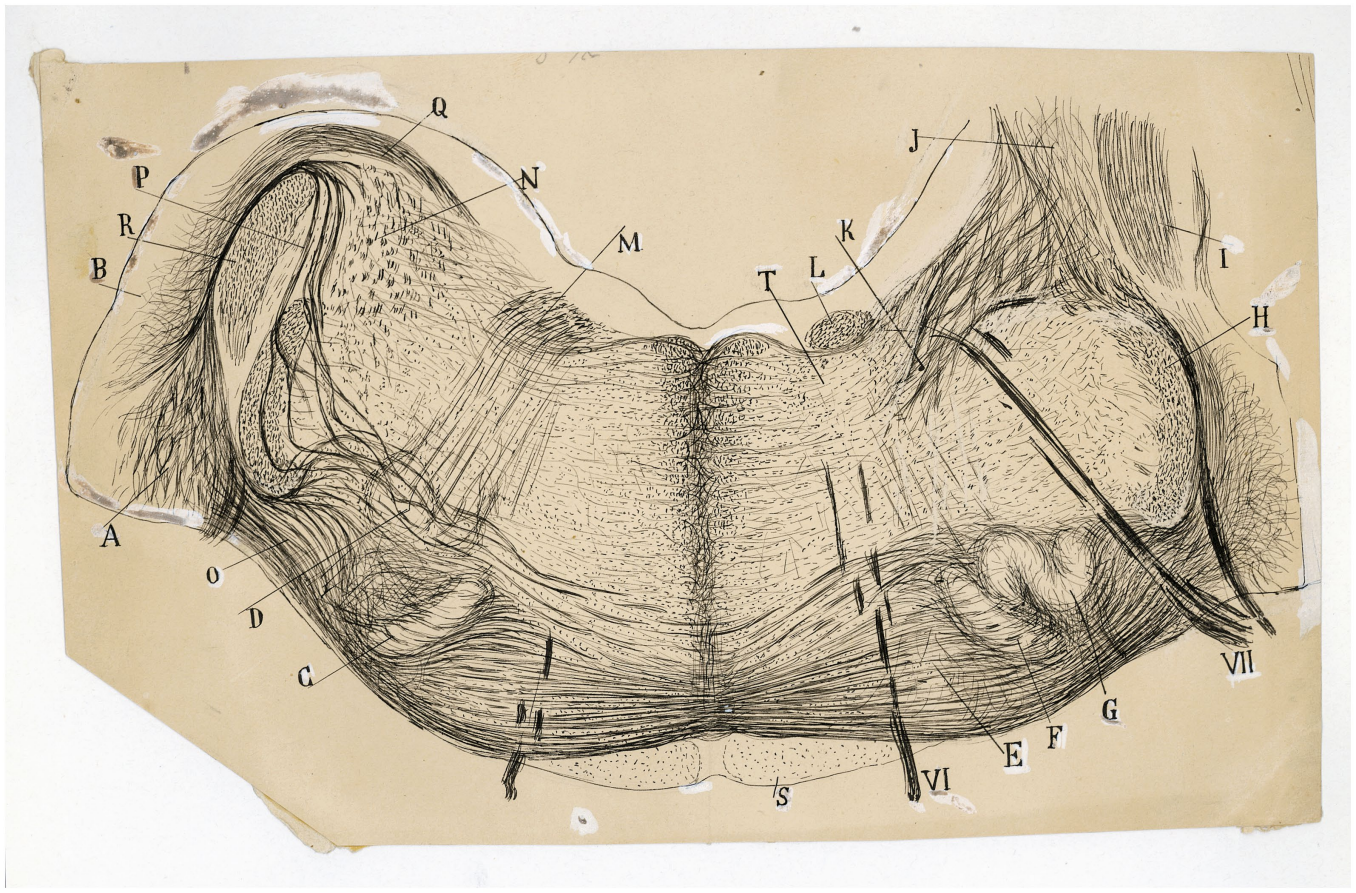
The fine organization around the trapezoid body in the cat. Frontal section through the medulla oblongata (P8 cat, Weigert-Pal method; the right half of the section is rostral is rostral to the left half). *(A)* Ventral and *(B)* dorsal cochlear nuclei; *(C,F)* medial superior olive; *(G)* lateral superior olive; *(D)* facial nucleus; *(E*) nucleus of the trapezoid body; *(H)* spinal root of the trigeminal nerve; *(I)* inferior cerebellar peduncle; *(J)* juxtarestiform body; *(K)* central pathway of the lateral vestibular nucleus; *(L)* ascending component of the facial root; *(M)* first inflection of this root; *(N)* lateral vestibular nucleus; *(O)* lateral and *(P)* intermediate trapezoid fibers; *(Q)* acoustic striae; *(R)* restiform body; *(S)* pyramidal tract; *(T)* abducens nucleus; *(VI)* abducens nerve, *(VII)* facial nerve. [Cajal’s original drawing, from Archivo científico Fernando de Castro. Madrid; first published in Ramón y Cajal, 1899–1904, vol. 2, Figures 219 and 285].

**Figure 10:**
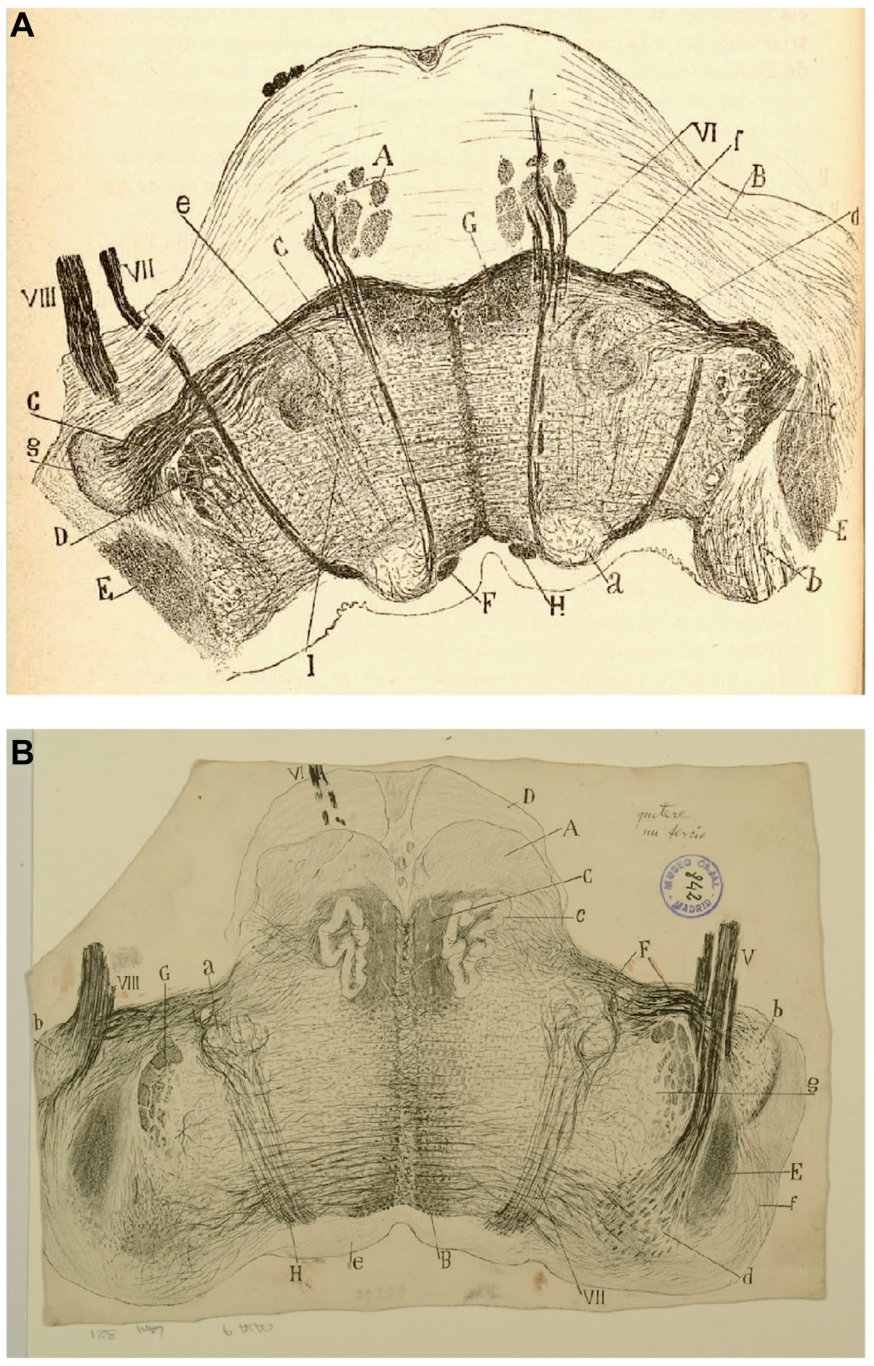
The fine organization of the pons in the newborn human. **(A)** Section ttrough the caudal fourth of the pons (newborn human, Weigert-Pal method). *(A)* Pyramids; *(B)* medial cerebellar peduncles; *(C)* trapezoid body; *(D)* trigeminal nucleus; *(E)* restiform body; *(F)* ascending portion of the facial nerve; *(G)* central sensitive pathway; *(H)* MLF; *(I)* part of the olivar complex; *(a)* nucleus of the external oculomotor nerve; *(b)* lateral (Deiters´) vestibular nucleus; *(c)* gelatinous substance of the trigeminal nerve; *(d)* superior olive; *(e)* accesory superior olive; *(f)* nucleus of the trapezoid body; *(g)* ventral cochlear nucleus; *(VI)* external oculomotor; *(VII)* facial and *(VIII)* statoacoustic cranial nerves [reproduced from Ramón y Cajal, 1899–1904, vol. 2, Figure 218]. **(B)** Section through the medulla at the posterior edge of the pons (P15 human, Weigert-Pal method). *(A)* pyramids; *(B)* MLF; *(C)* sensory pathway; *(D)* pons; *(E)* inferior cerebellar peduncle; *(F)* trapezoid body fibers; *(G)* descending root of the trigeminal nerve, *(H)* facial nerve fibers; *(a)* facial nerve nucleus, *(b)* ventral cochlear nucleus; *(c)* olive; *(d)* descending (inferior) vestibular nucleus; *(e)* central gray matter; *(f)* dorsal cochlear nucleus; *(g)* gelatinous subnucleus of spinal nucleus of trigeminal nerve; *(V)* vestibular nerve, *(VI)* abducens nerve, *(VII)* facial nerve; *(VIII)* cochlear nerve [Cajal’s original drawing, from Legado Cajal-CSIC, Madrid; first published in Ramón y Cajal, 1899–1904, vol. 2, Figure 217].

**Figure 11:**
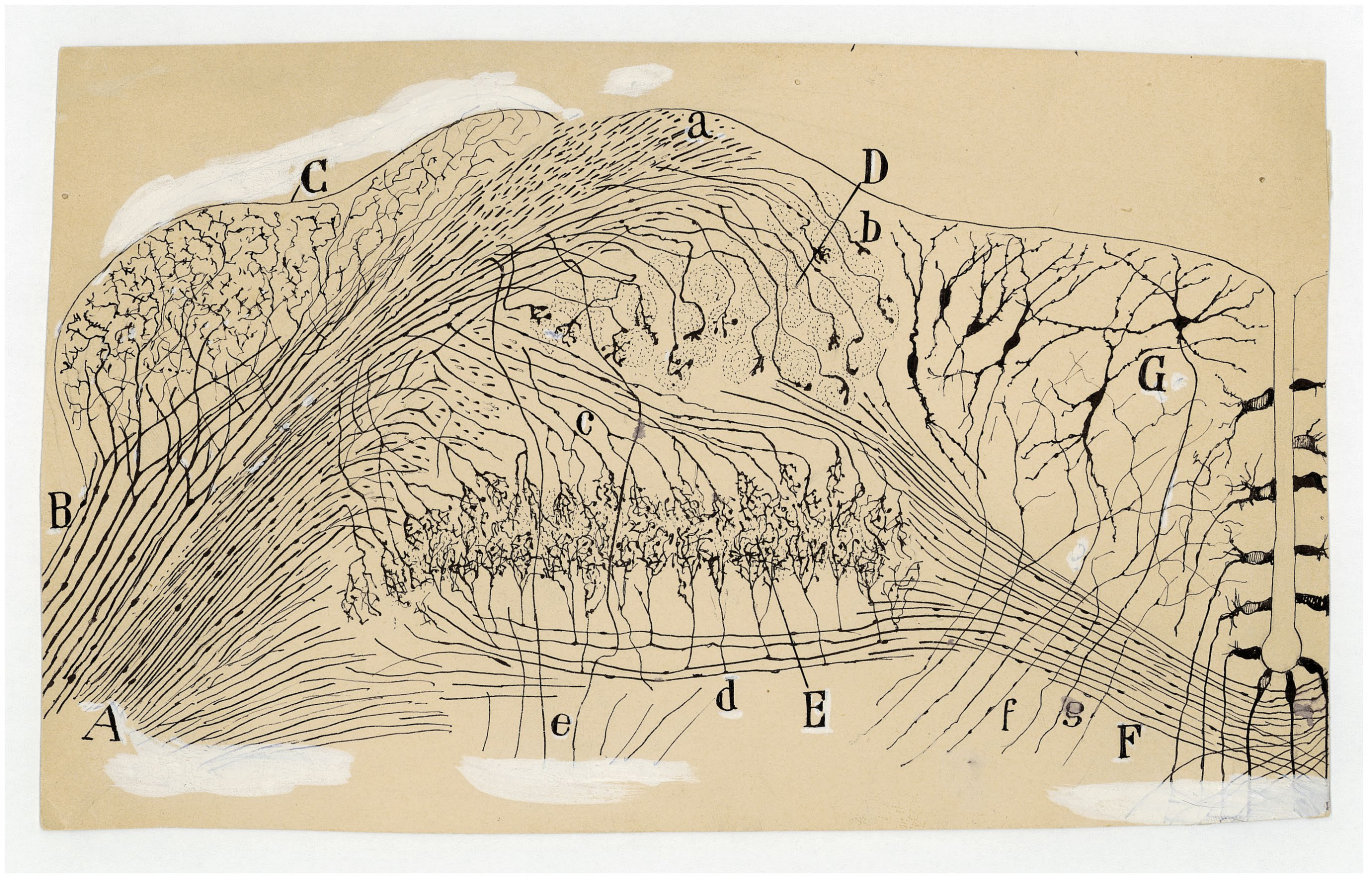
Auditory and vestibular nuclei (E14 chick embryo, Golgi method). *(A)* Radicular fibers of the cochlear nerve; *(B)* bifurcations of the radicular fibers; *(C)* angular nucleus; *(D)* magnocelullar nucleus; *(E)* parvocelullar nucleus; *(F)* trapezoid body; *(G)* medial vestibular nucleus; *(a)* obliquely sectioned ascending branches of cochlear nerve; *(b)* terminals bulbs of these fibers; *(c)* cochlear collaterals destined to the parvocelullar nucleus; *(d)* other collaterals destined to the same nucleus, but coming from the opposite side; *(e)* descending branch of bifuraction of cochlear fibers; *(f)* afferent vestibular fibers; *(g)* descending axon of medial vestibular nucleus neuron [Cajal’s original drawing, from Archivo científico Fernando de Castro. Madrid; first published in Ramón y Cajal, 1899–1904, vol. 2, Figure 289].

**Figure 12:**
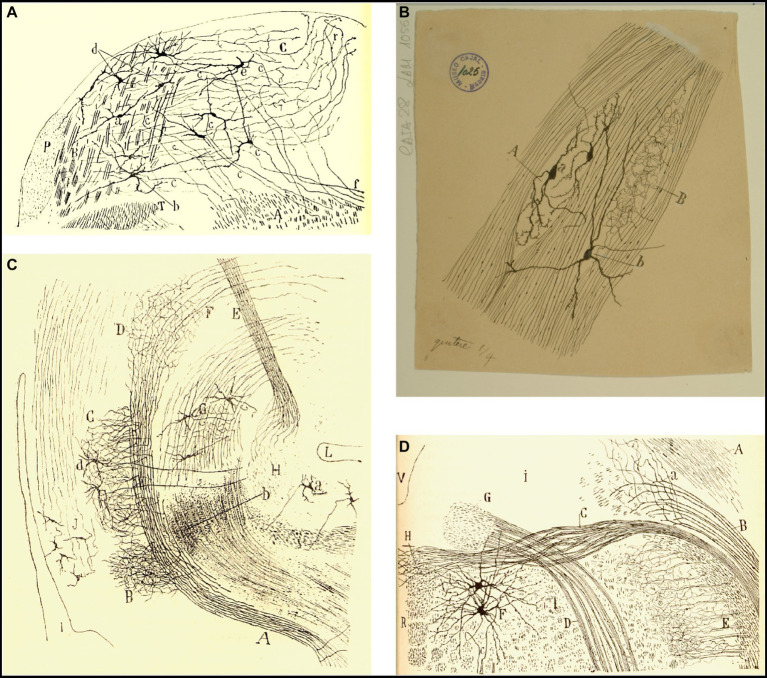
Cajal’s descriptions of the vestibular pathway in mammals during development. **(A)** Frontal section through the medulla, caudal to the genu of the facial nerve (P4 mouse, Golgi method). *(A)* Lateral reticular formation containing the ipsilateral (uncrossed) vestibular pathway, *(B)* lateral part of the spinal vestibular nucleus, *(C)* medial part of the spinal vestibular nucleus, *(P)* inferior cerebellar peduncle, *(T)* spinal root of the trigeminal nerve; *(a,b)* neurons whose axon courses toward the ipsilateral (uncrossed) vestibular pathway, *(c)* axons, *(d)* cells whose axons course laterally; *(e,f)* axons directed toward the raphé [reproduced from Ramón y Cajal, 1895b, Figure 20; also appeared in Ramón y Cajal, 1896, Figure 20, and in Ramón y Cajal, 1899–1904, vol. 2, Figure 252]. **(B)** Intersticial nuclei of the vestibular nerve (newborn kitten, Golgi method). *(A)* Cells; *(B)* collaterals; *(a)* neurons with short dendrites; *(b)* neurons with dendrites distributed in several nuclei [Cajal’s original drawing, from Legado Cajal-CSIC, Madrid; first published in Ramón y Cajal, 1899–1904, vol. 2, Figure 253]. **(C)** Saggital section through the MLF (E19 mouse embryo). *(A)* MLF at pons level; *(B)* collaterals from MFL to the trochlear nerve nucleus; *(C)* collaterals for the oculomotor nerve nucleus; *(D)* terminal arborizations from MLF to its nucleus; *(F)* MLF crossing Meinert’s bundle to entry in the thalamus; *(E)* Meynert’s retroflex bundle; *(G)* red nucleus; *(H)* interpeduncular nucleus; *(I)* posterior entry to Sylvius aqueduct; *(L)* pontomamillary sulcus ending; *(a)* interpeduncullar cell; *(b)* dorsal tegmental decussation fibers; *(d)* radicular fibers of oculomotor nerve; *(J)* central gray substance cells, with ascending axons [reproduced from Ramón y Cajal, 1895b, Figure 14]. **(D)** Transversal section of the medulla oblongata (kitten fetus, Golgi method). *(A)* Restiform body; *(B)* ipsilateral (uncrossed) vestibular pathway; *(C)* contalateral (crossed) vestibular pathway; *(D)* facial nerve; *(F)* abducens nucleus [reproduced from Ramón y Cajal, 1897, Figure 10; it also appeared in Ramón y Cajal, 1899–1904, vol. 2, Figure 319].

The vestibular nuclei are located immediately beneath the lateral border of the floor of the fourth ventricle. Based on their cytoarchitecture and using sections of mouse embryos and newborns stained with the method of double silver impregnation, Cajal characterized four vestibular nuclei: the Bechterew’s nucleus, the lateral or Deiters´ nucleus, the dorsal or principal nucleus and the descending nucleus (Ramón y Cajal, 1895b, pp. 65–72, 1896, pp. 7–75, 1899–1904, vol. 2.1, pp. 98–107). He painstakingly described them:

- The Bechterew’s nucleus is found dorsal to the Deiters´ nucleus, lateral to the lateral wall of the fourth ventricle, and medial at the limit of the inferior cerebellar peduncle; it contains numerous multipolar medium-sized neurons whose axons profusely collateralize within the nucleus itself before coursing ventrally to enter the lateral vestibular nucleus (Figure 6A).
- The nucleus of Deiters is located under the floor of the fourth ventricle, between the descending nucleus (medially) and the restiform body (laterally); its neurons are star-shaped and multipolar giant cells, with long spiny dendrites surrounded by fibers of the descending branch of the vestibular nerve, collaterals and terminal fibers often form so-called pericellular baskets (Figures 8, 9, 10A).
- The dorsal nucleus has a triangular shape in a cross-section, lies in the floor of the fourth ventricle, medial to, and in continuity with the lateral vestibular nucleus. According to Cajal, this can be distinguished from the Deiters´ one because it lacks bundles of thick fibers, and its neurons are smaller (triangular, and fusiform or stellate, and surrounded by collateral coming from the descending branch of the vestibular nerve (Figures 8A, 11).
- The descending nucleus lies dorsal to the spinal root of the trigeminal nerve and is characterized by small bundles of descending branches of the vestibular nerve along with small, fusiform or triangular cells, provided with long and varicose dendrites (Figures 10B, 12A). Cajal described vagal and glossopharyngeal afferent fibers projecting to the descending nucleus.

The morphology of the vestibular nuclei was later detailed by Lorente de Nó (1933a) and especially clarified by Brodal and Pompeiano in the 1950s, distinguishing four main vestibular nuclei in the cat: superior (of Bechterew), medial (of Schwalbe), lateral (of Deiters), and inferior, and several minor cell groups (Brodal and Pompeiano, 1957). Among these groups is the interstitial nucleus of the vestibular nerve, which was initially described by Ramón y Cajal (1903, 1899–1904, vol. 2.1, pp. 103–104) (Figure 12B). This small nucleus is found before the vestibular nerve reaches the lateral vestibular nucleus and divides into ascending and descending roots. Although Cajal recognized the difficulty to find homologies between the vestibular nuclei in birds and mammals, he suggested that this interstitial nucleus, which is so poorly developed in mammals, may correspond to the large tangential nucleus that he discovered in fishes and birds (Ramón y Cajal, 1908b; 1908d). In addition, the cerebello-acusticus nucleus reported by Ramón y Cajal (1895b, p. 65; 1896, p. 66, 1899–1904, vol. 2.1, p. 98; 1908c) seems to correspond to what we currently know as group “y” (Brodal, 1974).

It is important to highlight that both Ramón y Cajal (1895b) and Lorente de Nó (1933b) did not observe a significant number of true Golgi type II interneurons in the vestibular nuclei. Hauglie-Hanssen (1968), also using Golgi staining, did not find this type of neurons in the vestibular nuclei either. However, other authors found interneurons in commissural pathways (Shimazu and Precht, 1966).

### The efferent pathways from the vestibular nuclei

The main efferent projections from the vestibular nuclei are the vestibulocerebellar pathways, medial longitudinal fascicle (MLF), medial vestibulospinal tract, lateral vestibulospinal tract, and vestibulothalamic pathways, as well as connections with the contralateral vestibular nuclei through the commissural pathways.

According to Cajal, the axons of the neurons in the vestibular nuclei are organized into two separate second-order vestibular pathways: (i) one is the lateral, direct, or ipsilateral pathway, which was initially described by Held and is a longitudinal tract arising from the medial and lateral vestibular nucleus that runs ventrolateral to the abducens nucleus (Held, 1892, 1893), (ii) the other is the medial or crossed pathway, which constitutes a significant portion of the contralateral MLF (Figures 7B, 8). Cajal was the first author to appreciate this medial or crossed pathway that contributes to the MLF in mammals (Ramón y Cajal, 1895b, p. 69, 1899–1904, pp. 105–107).

He also extensively described the ascending fibers of the MLF, detailing its main origin in the Deiters´ nucleus and how them reach the nuclei of cranial nerves III, IV, and VI, as well as the Edinger’s nucleus (Ramón y Cajal, 1895b, pp. 1–59, 1899–1904, vol. 2, pp. 547–554; Figures 8A, 12C). Notwithstanding, we now know that the main ascending tracts originate from the superior and medial vestibular nuclei (McMaster et al., 1966). On the other hand, Cajal specifically highlighted the role of the second-order vestibular neurons within the MLF in generating compensatory eye movements after changing the position of the head or body (Ramón y Cajal, 1895b, pp. 59, 1899–1904, vol. 2, p. 162). The current knowledge interprets the MLF as the ultimate pathway for all types of conjugate eye movements, including saccades, smooth pursuit, and the vestibulo-ocular reflex (Leigh and Zee, 2015).

**Figure 13:**
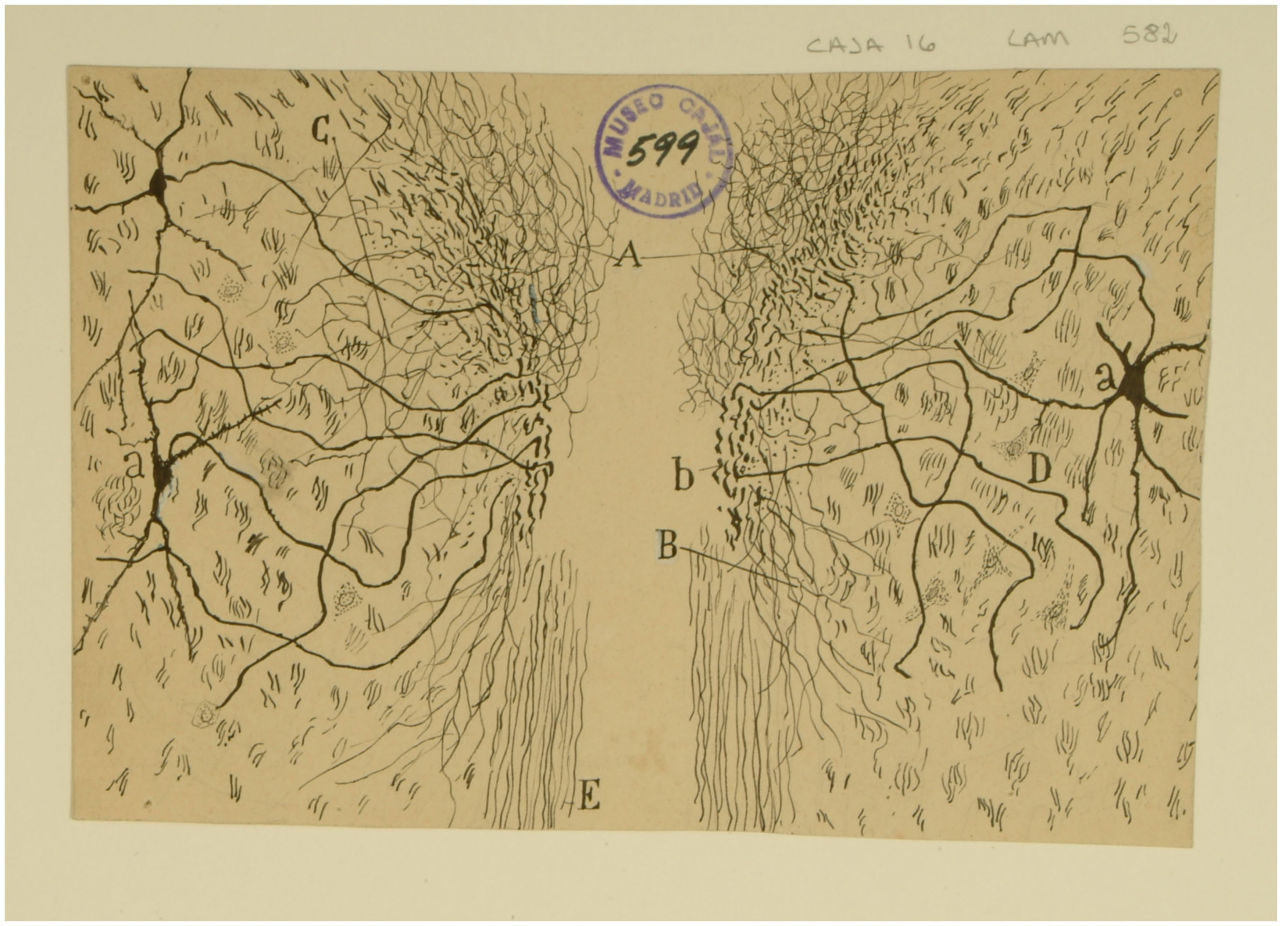
The interstitial nucleus of Cajal. Frontal section through the tegmentum just rostral to the red nucleus (newborn mouse, Golgi method). *(A)* Plexus of collaterals and parent fiber terminals associated with the MLF; *(B)* descending collaterals of the MLF; *(C)* lateral collaterals of the MLF; *(D)* interstitial nucleus; *(a)* cells of the interstitial nucleus; *(b)* region of the MLF where axons of the interstitial nucleus are concentrated [Cajal’s original drawing, from Legado Cajal-CSIC, Madrid; first published in Ramón y Cajal, 1899–1904, vol. 2, Figure 505].

In addition, it is important that Cajal also depicted the interstitial nucleus of the MLF, which is currently known as to the interstitial nucleus of Cajal (INC) or accessory oculomotor nucleus (Ramón y Cajal, 1895b, p. 1,^1899–1904^, vol. 2.2, p. 551, 1908a; Figure 13). The INC is a prominent cell group situated within the fibers of the MLF, lateral to the rostral pole of the oculomotor nucleus, ventrolateral to the periaqueductal gray and immediately caudal to the rostral interstitial nucleus of the MLF (riMLF). Cajal pointed out its participation in the reflex pathway that connects the vestibular nuclei with the oculomotor nuclei (Ramón y Cajal, 1909–1911, vol. 2, p. 263–269). Nowadays, its projections towards vestibular nuclei are well known as well as with the paramedian pontine reticular formation, several thalamic nuclei, and the ipsilateral oculomotor and trochlear nuclei. The INC participates, alongside the riMLF, in the generation of vertical gaze and torsional eye movements (Leigh and Zee, 2015).

As for the prepositus hypoglossal nucleus (nucleus prepositus hypoglossi, NPH), Cajal only mentioned the existence of “borderline” cells of the hypoglossal nucleus and that they would not be related to the motor function of this cranial pair. At present, we know that the NPH is involved in the generation of horizontal conjugate eye movements.

Cajal also confirmed the existence of a vestibulospinal projection made up of descending axonal branches from the ipsilateral lateral nucleus and indicated that probably also from the descending nucleus (Ramón y Cajal, 1895b, 1899–1904, vol. 2, pp. 102–107). Equally, he pointed that the inferior cerebellar peduncle contains other pathways that arise in the vestibular region and may end in the spinal cord (Ramón y Cajal, 1899–1904).

On the other hand, he also described the existence of a crossed vestibular bundle, although he was unable to prove that it terminated in the opposite vestibular nuclei (Ramón y Cajal, 1897; Figure 12D). Currently, we know that the commissural fibers interconnect the vestibular nuclei of the two sides, except for lateral vestibular nuclei (Holstein, 2012).

Although Cajal described the bidirectional connections between the vestibular nuclei and the pontomedullar reticular formation (Ramón y Cajal, 1895b, pp. 58–59, 1896, pp. 59–60, 1899–1904, vol. 2.1, pp. 106–107), it was Lorente de Nó who better established the role of the reticular formation in the mechanisms of the vestibulo-ocular reflex (Lorente de Nó, 1933b; Espinosa-Sanchez et al., 2023). Collaterals of the vestibulospinal tract to the reticular formation were described by Cajal. We have not found that Cajal pointed out the existence of vestibulothalamic pathways that are known today.

## Physiology of the vestibular system

**Figure 14:**
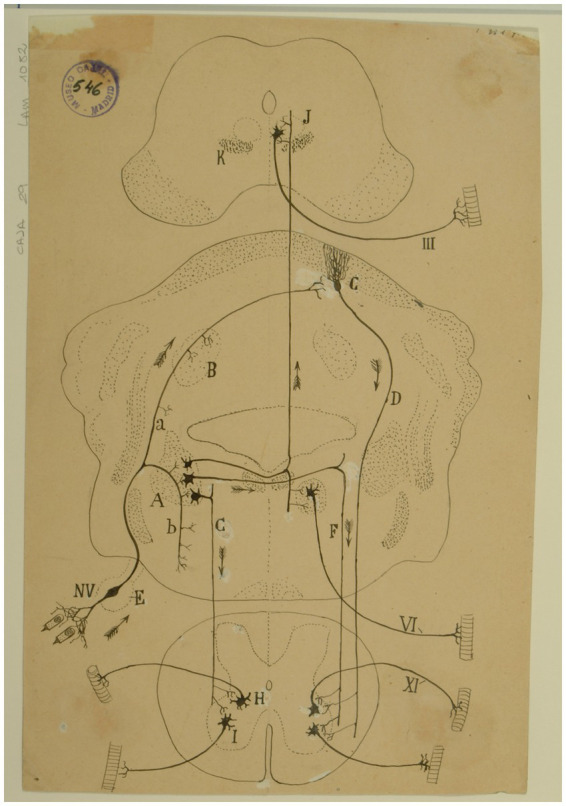
Diagram of the sense followed by the excitations of the vestibular pathways of the cat (Arrows indicate the direction of impulses). *(A)* Lateral (Deiters´) vestibular nucleus, *(B)* fastigial nucleus, *(C)* Purkinje cells, *(D)* Marchi’s descending pathway, *(E)* Scarpa’s ganglion, *(F,G)* short vestibular pathways of the medulla, *(H)* spinal nucleus of the 11^th^ cranial nerve, *(I)* motor nuclei of cervical nerves, *(J)* oculomotor nucleus, *(K)* MLF, *(III)* oculomotor nerve, *(VI)* abducens nerve, *(XI)* spinal accesory nerve, *(NV)* vestibular nerve [Cajal’s original drawing, from Legado Cajal-CSIC, Madrid; first published in Ramón y Cajal, 1899–1904, vol. 2, Figure 287].

At the end of each chapter of his *Textura*, Cajal used to do some physiological comments. Regarding the vestibular system, in addition to presenting a scheme of the vestibular system (Figure 14), he offers us a fairly current definition of the vestibulo-spinal and vestibulo-ocular reflex:

El impulso recolectado por el grupo de bipolares anejas a un conducto semicircular, se propagará directamente a la lámina cerebelosa en donde moran las células de Purkinje, que rigen los focos motores (mediante la vía descendente de Marchi, de la medula), bulbares y medulares inervadores de los músculos que realizan la acción equilibradora.
La rama descendente y sus colaterales, así como las vías secundarias engendradas en los núcleos de Deiters, dorsal, etc., podrían servir para producir movimientos asociados y conjugados de orden reflejo. Así, por ejemplo: mediante la participación que el núcleo de Deiters tiene en la construcción del fascículo longitudinal posterior (relacionado, como es sabido, con los núcleos motores de los ojos), se explicaría fácilmente este hecho: que, cuando moviendo la cabeza, miramos un objeto, los ojos giran en sentido inverso de esta, a fin de mantener la imagen en la foseta central (Ramón y Cajal, 1899–1904, vol. 2, pp. 161–162).
[The impulse collected by the group of bipolar cells innervating a particular semicircular canal, would propagate directly to the cerebellar lamina containing the Purkinje cells that influence bulbar and spinal motor nuclei (by way of the descending pathway of Marchi of the medulla) innervating muscles that maintain equilibrium.
The descending branch and its collaterals, as well the secondary pathways generated in the lateral, medial nuclei, etc., could serve to produce associated and conjugate movements of reflex nature. Thus, for example, through the contribution of the lateral vestibular nucleus to the medial longitudinal fasciculus, related as it is known to oculomotor nuclei, the following fact could be easily explained. When we look at an object while moving the head, the eyes deviate in the opposite direction to maintain the image on the fovea.]

Finally, in that chapter, Cajal pointed out that the sense of nerve impulses along the vestibular pathway is a good claim in support of the “principle of dynamic polarization of neurons” and the concept of “conduction avalanche,” which was also proposed by him in 1895 (Ramón y Cajal, 1895a).

**Figure 15:**
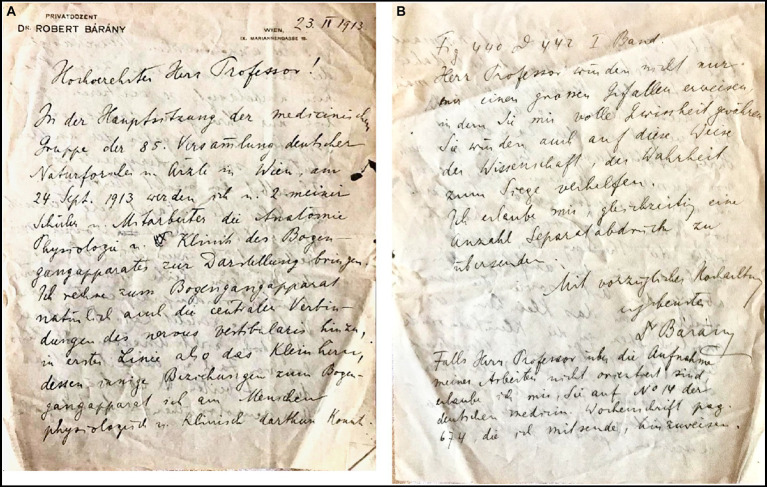
Letter from Robert Bárány to Santiago Ramón y Cajal, dated in Vienna, on February 23rd, 1913. To our knowledge, this is the first letter ever interexchanged between Barany and Cajal. **(A)** First page of the letter. **(B)** Fourth and last page of the letter. [This original letter is written in German and is part of the Archivo científico Fernando de Castro, Madrid (Spain)].

The Austrian otologist Robert Bárány (1876–1936) published in 1907 his seminal work in which he thoroughly explained the mechanism of caloric nystagmus (Bárány, 1907). This is the conjugated eye movement that appears after irrigating the ear with hot and cold water, which is the basis of the caloric test that we still perform today to evaluate vestibular function. In 1913, Bárány prepared a lecture on the function of the semicircular canals for the 85^th^ Meeting of the Society of German Naturalists and Physicians (Bárány, 1913). To include additional relevant visual support, he contacted Cajal and requested two prepared microscope slides with sections featured in the “Histologie du système nerveux de l’homme et des vertébrés” (Figure 15). Specifically, Bárány requested slides that displayed the ascending and descending roots of the vestibular nerve as well as the vestibulo-cerebellar fibers (among them, Figures 5A, 6A). This correspondence is significant because it demonstrates that Bárány already recognized Cajal’s authority in the field, acknowledging that only Cajal’s precise data were truly reliable: “only your very precise data exist. The data from other authors are debatable.”^2^ The following year, in 1914, the Nobel Committee for Physiology or Medicine evaluated the nominations for that year’s prize and determined that none of them met the established criteria. Consequently, in compliance with the Nobel Foundation’s statutes, the committee decided to reserve the prize for the following year. A year later, in 1915, Robert Bárány was awarded the Nobel Prize corresponding to 1914.

## Final remarks

Delving into Cajal’s work reveals a wealth of undiscovered gems. For instance, his ideas on CNS plasticity and neurogenesis are instrumental in elucidating the mechanisms of vestibular compensation following vestibular injury, something that we are just beginning to understand. Cajal’s contributions to vestibular anatomy and histology are remarkable. He detailed the sensory nerve endings, anatomy of the vestibular nerve and Scarpa ganglion, afferent vestibular fibers, vestibular nuclei, lateral vestibulospinal tract, vestibulocerebellar connections, and the intricate structure of the cerebellum. These findings, together with the staining methods developed by him and his disciples, benefited subsequent investigators. Among these scholars stands out the figure and work of Rafael Lorente de Nó, his last and youngest disciple, who laid the groundwork for our current understanding of the vestibulo-ocular reflex. Cajal’s contributions make him worthy of recognition as a founder of vestibular science.

## Footnotes

^1^Letter from Ramón y Cajal to Retzius. Madrid, December 10, 1892. Ref. 21,905,454,012-13/460/Fotocopias 1–2. Instituto Cajal, Madrid (Spain).

^2^Letter from R. Bárány to SR Cajal. Wien, Feb 23, 1913. Archivo científico Fernando de Castro (Censo-Guía de Archivos de España e Iberoamérica #ES.28079.AFC, Madrid -Spain-, included by UNESCO in the Memory of the World International Register of the Human Heritage, as “Archives of Santiago Ramón y Cajal and the Spanish Neurohistological School” collection ID 2016-312,016).
